# Multimodal MRI study on the relation between WM integrity and connected GM atrophy and its effect on disability in early multiple sclerosis

**DOI:** 10.1007/s00415-023-11937-2

**Published:** 2023-09-17

**Authors:** Merlin M. Weeda, D. R. van Nederpelt, J. W. R. Twisk, I. Brouwer, J. P. A. Kuijer, M. van Dam, H. E. Hulst, J. Killestein, F. Barkhof, H. Vrenken, P. J. W. Pouwels

**Affiliations:** 1 MS Center Amsterdam, Radiology and Nuclear Medicine, Vrije Universiteit Amsterdam, Amsterdam Neuroscience, Amsterdam UMC Location VUmc, Amsterdam, The Netherlands; 2 Epidemiology and Data Science, Amsterdam UMC Location VUmc, Amsterdam, The Netherlands; 3 MS Center Amsterdam, Anatomy and Neurosciences, Vrije Universiteit Amsterdam, Amsterdam Neuroscience, Amsterdam UMC Location VUmc, Amsterdam, The Netherlands; 4 Health-, Medical-, and Neuropsychology Unit, Institute of Psychology, Leiden University, Leiden, The Netherlands; 5 MS Center Amsterdam, Neurology, Vrije Universiteit Amsterdam, Amsterdam Neuroscience, Amsterdam UMC Location VUmc, Amsterdam, The Netherlands; 6 UCL Queen Square Institute of Neurology and Centre for Medical Image Computing, University College London, London, UK

**Keywords:** Multiple sclerosis, MRI, White matter damage, Atrophy, Connectivity

## Abstract

**Background:**

Multiple sclerosis (MS) is characterized by pathology in white matter (WM) and atrophy of grey matter (GM), but it remains unclear how these processes are related, or how they influence clinical progression.

**Objective:**

To study the spatial and temporal relationship between GM atrophy and damage in connected WM in relapsing–remitting (RR) MS in relation to clinical progression.

**Methods:**

Healthy control (HC) and early RRMS subjects visited our center twice with a 1-year interval for MRI and clinical examinations, including the Expanded Disability Status Scale (EDSS) and Multiple Sclerosis Functional Composite (MSFC) scores. RRMS subjects were categorized as MSFC decliners or non-decliners based on ΔMSFC over time. Ten deep (D)GM and 62 cortical (C) GM structures were segmented and probabilistic tractography was performed to identify the connected WM. WM integrity was determined per tract with, amongst others, fractional anisotropy (FA), mean diffusivity (MD), neurite density index (NDI), and myelin water fraction (MWF). Linear mixed models (LMMs) were used to investigate GM and WM differences between HC and RRMS, and between MSFC decliners and non-decliners. LMM was also used to test associations between baseline WM *z*-scores and changes in connected GM *z*-scores, and between baseline GM *z*-scores and changes in connected WM *z*-scores, in HC/RRMS subjects and in MSFC decliners/non-decliners.

**Results:**

We included 13 HCs and 31 RRMS subjects with an average disease duration of 3.5 years and a median EDSS of 3.0. Fifteen RRMS subjects showed declining MSFC scores over time, and they showed higher atrophy rates and greater WM integrity loss compared to non-decliners. Lower baseline WM integrity was associated with increased CGM atrophy over time in RRMS, but not in HC subjects. This effect was only seen in MSFC decliners, especially when an extended WM *z*-score was used, which included FA, MD, NDI and MWF. Baseline GM measures were not significantly related to WM integrity changes over time in any of the groups.

**Discussion:**

Lower baseline WM integrity was related to more cortical atrophy in RRMS subjects that showed clinical progression over a 1-year follow-up, while baseline GM did not affect WM integrity changes over time. WM damage, therefore, seems to drive atrophy more than conversely.

## Introduction

In multiple sclerosis (MS), the most common pathological brain changes are widespread pathology in the white matter (WM) and atrophy of the grey matter (GM) [1]. Although GM atrophy shows stronger associations with clinical dysfunction than WM atrophy [2], large variability between patients’ atrophy rates and disability progression have been reported [3, 4]. It has been suggested that this inter-patient variability of GM atrophy rates arises to a large part because of the different distribution and severity of WM damage between patients, indicating the importance of looking at the GM–WM relationship in anatomically connected regions [5–8]. However, most studies investigating this had a cross-sectional design and could therefore not draw any conclusions on whether GM atrophy precedes of follows WM damage, and how this effects disability progression in MS.

A recent systematic review by Lie et al. [9] that included 90 studies on the relationship between WM lesions and GM volume showed an inverse association between the 2, particularly in early (relapsing) MS, and less so in progressive MS, suggesting that GM neurodegeneration is mostly secondary to WM damage in the form of lesions in early stages of the disease. Still, a knowledge gap is present since WM damage in MS is not confined to focal lesions, but to microstructural damage as well. Microstructural damage in the WM can be assessed indirectly through quantitative magnetic resonance (MR) measures, such as fractional anisotropy (FA), mean diffusivity (MD), axial diffusivity (AD) and radial diffusivity (RD), which are simplified measures obtained from the diffusion tensor for the overall integrity of the WM [10]. Multi-shell diffusion weighted imaging (DWI) can provide more biophysical properties such like neurite orientation dispersion and density imaging (NODDI) [11], thereby providing more information on microstructural damage in the WM. Recent studies have shown that NODDI parameters such as neurite density index (NDI) and orientation dispersion index (ODI) enable additional characterization of the WM in MS subjects [12, 13]. In particular, NDI can be considered an axonal marker [14]. Other imaging techniques that can be used for further characterization of damage in the WM are quantitative susceptibility mapping (QSM) and myelin water imaging (MWI). QSM may enable us to visualize WM damage and lesion formation before conventional structural imaging could [15], and from MWI, the myelin water fraction (MWF) can be calculated, which can provide information on the myelin content of the WM [16]. Combining these imaging measures may enable us to visualize more subtle WM damage patterns that occur in the early stages of the disease.

In this study, we aimed to investigate whether in early RRMS, the amount and/or type of damage in the WM tracts connected to the GM influences neurodegeneration, or whether damage in the GM influences damage in the connected WM tracts over time, and how this may relate to disability. For this, we studied longitudinal multimodal imaging data to better understand the spatiotemporal relationship between WM and GM damage in early RRMS.

## Methods

### Subjects

The institutional review board approved the study protocol and all participants gave written informed consent prior to participation, according to the Declaration of Helsinki. To be included in the study, patients had to be diagnosed with clinically definite RRMS [17] and have a disease duration of no more than 5 years. They could be included if they were using no treatment or first-line treatment, but not if they were using more advanced treatment. Their clinical disability levels had to be limited, with a maximum allowed EDSS score of 5.0. In case of switching of treatment, MRI examinations were planned with at least 4–6 months of delay [18]. When steroids were used, MRI was delayed by 3 months [19]. Patients were excluded (over the course of the study) in case of (switching to) second-line treatment to avoid spurious (pseudo)atrophy effects. Patients were seen at 1-year intervals, for extensive MR imaging and evaluation of clinical and neuropsychological performance.

A group of age-, gender- and education-matched healthy controls (HCs) was also included and seen at 1-year intervals for extensive MR imaging. Exclusion criteria for both MS and HC subjects were inability to undergo MRI examination; and past or current clinically relevant neurological, psychiatric or (auto)immune disorders other than MS. The data used in the current study were part of a larger cohort study, some results of which have been reported previously [20].

### MR imaging—acquisition

This was a single-center study in which a single MR scanner was used without intermediate upgrades. Imaging was performed on a 3 T whole-body scanner (Discovery MR750, GE Healthcare, Milwaukee, WI., USA) and an eight-channel phased-array head coil. The MR protocol included a sagittal 3D T1-weighted fast spoiled gradient echo sequence (FSPGR with TR/TE/TI = 8.2/3.2/450 ms and 1.0 mm isotropic resolution) and a sagittal 3D T2-weighted fluid attenuated inversion recovery sequence (FLAIR with TR/TE/TI = 8000/130/2338 ms at resolution 1.0 × 1.0x1.2 mm).

Axial 2D DWI acquisitions covering the entire brain (echo planar imaging, TR/TE = 6200/86 ms with 2.0 mm isotropic resolution) with accompanying reference scans with reversed phase-encoding direction were performed. The multi-shell DWI consisted of 7 volumes without diffusion weighting (*b* = 0 s/mm^2^) and 88 volumes with non-colinear diffusion gradients (29 images with *b* = 500 s/mm^2^ and 59 images with *b* = 2000 s/mm^2^). The b-values were interleaved to improve post-processing steps like motion correction [21].

Quantitative susceptibility mapping (QSM) images were acquired using an axial 3D multiple echo GRE sequence, with 7 TEs (TR/TEstart/TEdelta/TEmax = 49.24/5.44/6.55/44.73 ms at resolution 0.5 × 0.5 × 1.6 mm).

Lastly, multicomponent driven equilibrium single pulse observation of T1 and T2 (mcDESPOT) was acquired in an axial 3D slab with 2.5 mm isotropic resolution, using a spoiled gradient recalled sequence (SPGR with TR/TE = 8/3.02 ms with multiple flip angles [3°, 4°, 5°, 6°, 7°, 9°, 13°, 18°]), a balanced steady-state free precession sequence (SSFP with TR/TE = 4.736/2.368 ms, with multiple flip angles [12°, 16°, 21°, 27°, 33°, 40°, 51°, 65°], with phases 0° and 180° for the odd flip angles and phases 90 and 270 degrees for the even flip angles), and a 2D axial B1 map (TR/TE = 17/12.8 ms at lower resolution 4.0 × 4.0 × 5.0 mm). High-order shimming was performed for multi-shell DWI, QSM, and mcDESPOT acquisitions.

### MR imaging—analysis

**Fig. 1:**
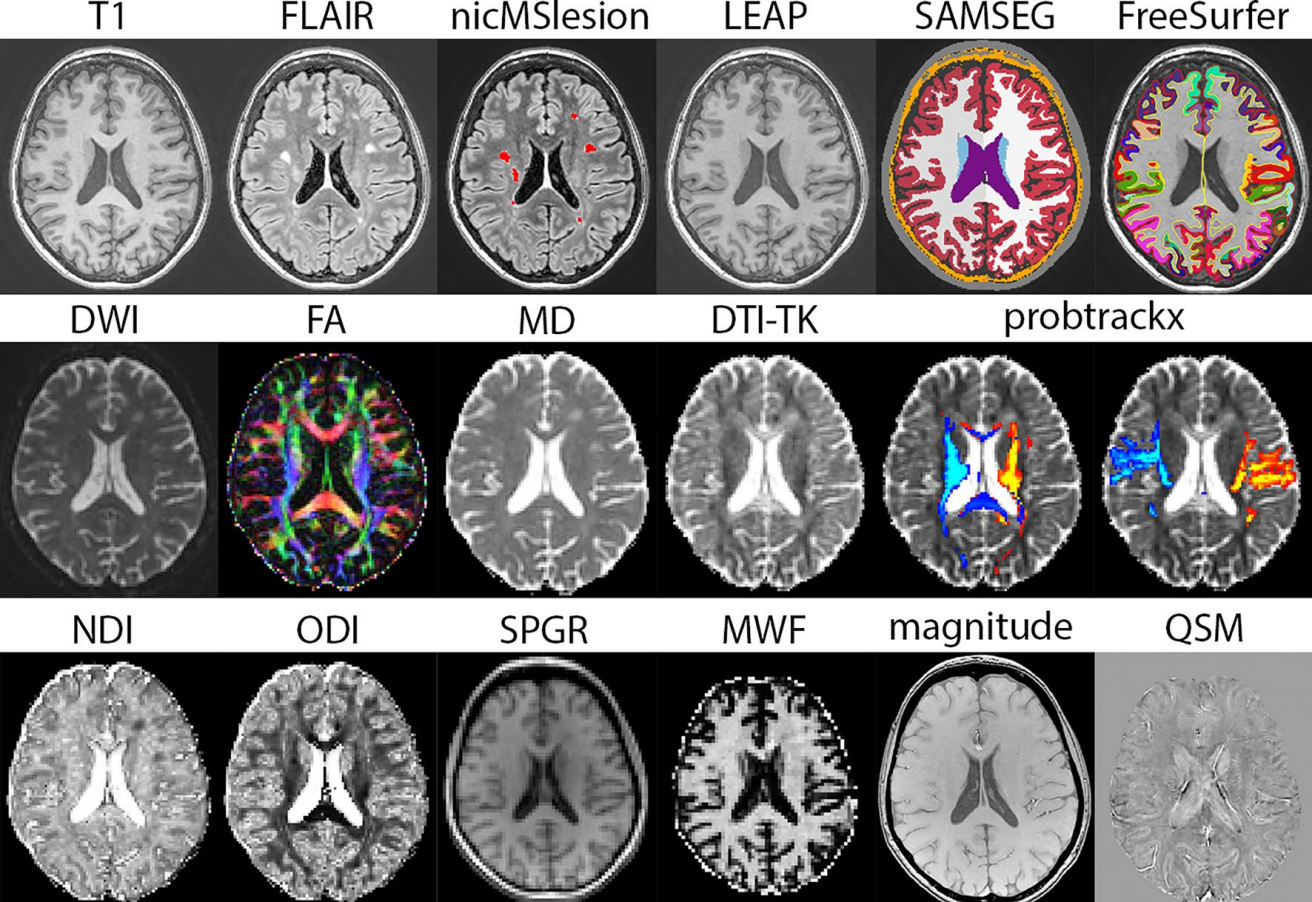
Overview of MR imaging analysis. Top row: T1 and FLAIR images were used to create a lesion mask (nicMSlesions) and lesion filled (LEAP) image, which was used as input for longitudinal SAMSEG for subcortical segmentation and longitudinal FreeSurfer for cortical parcellation. Middle row: multi-shell diffusion weighted images (DWI) were used to obtain FA and MD in the WM mask. A tensor image from DTI-TK was used to register FreeSurfer ROIs, and probtrackx was used for tractography (example from thalamus tracts and postcentral tracts). Bottom row: NODDI was used to obtain NDI and ODI maps; SPGR images were used to create MWF maps; magnitude and phase (not shown) images were used to create QSM maps

An overview of the different MRI post-processing streams used in this study is shown in Fig. 1 and analyses are described below.

#### Structural imaging analysis

Brain extraction was performed using FMRIB Software Library (FSL) version 5.0.10 brain extraction tool BET [22, 23] and N3 bias field correction with 3T optimized parameters was performed with FreeSurfer version 6.0 [24]. Lesion segmentation was performed with the deep-learning algorithm nicMSlesions version 0.2 [25, 26] which was optimized for our data in an earlier study [27]. In summary, full re-training of the nicMSlesions neural network (11 layers) was done with the use of manual lesion segmentations available from fourteen subjects with MS. All parameters were set at default. On the resulting lesion probability map, the optimized probability threshold of 0.4 was applied and lesions smaller than five voxels were removed.

Lesion filling was performed with LEsion Automated Processing (LEAP) [28] and lesion filled images were processed with the longitudinal pipeline of FreeSurfer [29–31], using a template-driven approach to provide a detailed parcellation and segmentation of the cortex and subcortical structures [32, 33]. Cortical thickness was obtained from FreeSurfer and brain volumes were obtained from the longitudinal pipeline of Sequence Adaptive Multimodal SEGmentation (SAMSEG) [34, 35]. To control for differences in head size, whole brain (WB), WM and DGM brain volumes were normalized and calculated as a percentage of the segmentation-based total intracranial volume (normalized WB [nWB], normalized WM [nWM], and normalized DGM [nDGM], respectively). In addition, nDGM volume and CGM thickness were converted into *z*-scores for each structure separately, based on the entire cohort. In our analysis, two DGM structures (nucleus accumbens and amygdala) and three CGM structures (entorhinal cortex, frontal pole and temporal pole) were excluded from analyses due to their known measurement variabilities [36].

#### Diffusion imaging analysis

All images were corrected for susceptibility induced geometric distortions using FSL topup [37, 38], and for movement and eddy currents with FSL eddy [39]. The diffusion tensor was fitted on all *b* = 0 mm/s^2^ and *b* = 2000 s/mm^2^ images to obtain the FA, MD, AD and RD values.

The multi-shell ball and stick model of bedpostx [40] was used to estimate the voxel-wise diffusion parameter distribution. Probabilistic tractography using 5000 streamlines was performed with probtrackx2 [41–43]. After linear registration of DWI to 3DT1, the inverse transformation was used to register the FreeSurfer regions of interest (ROIs) to DWI space using nearest-neighbour interpolation; thus per hemisphere, 5 DGM and 31 CGM regions were available as seed and target ROIs for tractography. A midline exclusion mask was used to ensure tracts could not cross hemispheres, except through the corpus callosum, fornix and brainstem. Moreover, the target ROIs were used as both waypoint and termination masks.

Probabilistic tractography was performed on year-1 scans, after which the tracts were propagated to the year-2 scans using Diffusion Tensor Imaging ToolKit (DTI-TK) version 2.3.1 [44, 45], to create a spatially normalized tensor within-subjects template. DTI-TK makes use of an affine registration algorithm with explicit tensor reorientation optimization [46]. The longitudinal pipeline as proposed by Keihaninejad et al*.* [47] was used. A DTI-TK within-subject template was created for each subject from the *b* = 2000 mm/s^2^ images. With these templates, the year-1 probabilistic connectivity distribution was registered to year-2 images for each subject.

Neurite Orientation Dispersion and Density Index (NODDI) Matlab Toolbox version 1.0.1 was used to obtain the neurite density index (NDI) and orientation dispersion index (ODI). Voxels for which the NODDI model failed (i.e. all voxels with *f*_iso_ > 0.99; *f*_icvf_ > 0.99; kappa = 0.05; and all error-code voxels) [11] were filtered out to create a tissue mask image to apply to the NDI and ODI images.

#### QSM imaging analysis

SuscEptibility mapping PIpeline tool for phAse image (SEPIA) version 0.7.2 [48] containing the Susceptibiltiy Tensor Imaging (STI) Suite [49] in MATLAB (The MathWorks, Natick, MA) was used for processing of magnitude and phase images after applying a brain mask obtained with FSL BET. After phase unwrapping with the optimum weights-laplacian-based method and background field removal with variable radius kernel, QSM images were estimated with iterative sparse linear equation and least square (iLSQR) with susceptibility of the whole brain defined as 0 parts per million (ppm). QSM maps were registered to DWI space of the corresponding time point.

#### Myelin water imaging analysis

From B1, SPGR and SSFP images, myelin water fraction (MWF) maps were estimated using qimcdespot (from Quantitative Imaging Tool [QUIT] [50] version 2.0.2), assuming a three-component model with exchange. Upper and lower bounds were slightly adapted based on inspection of histograms and in-house optimization within a group of HC subjects. MWF maps were registered to DWI space of the corresponding time point.

#### WM measures in probabilistic tracts

The probabilistic tracts were multiplied by the binary WM mask, and for all measures (i.e. FA, MD, AD, RD, NDI, ODI, MWF and QSM), mean values per tract were extracted, weighted by the connectivity probability to emphasize the tract center, and decrease the effect of spurious tracts. We determined all WM measures for the whole tract, consisting of normal-appearing (NA)WM and lesional WM, but also perilesional WM.

In order to combine WM measures, *z*-scores were calculated per tract for each WM measure, based on the entire cohort, and *z*-scores were combined depending on which information was present. We calculated a diffusion *z*-score (WM-Diffusion = (zFA-zMD-zRD)/3) as well as an extended WM *z*-score (WM-Extended = (*z*FA – *z*MD – *z*RD + *z*NDI + *z*MWF)/5), when data were available.

### Clinical and neuropsychological evaluation

Patients’ medical history was taken, including the occurrence of relapses and changes in therapy. Neuropsychological evaluation included the Symbol Digit Modalities Test (SDMT) [51] to measure information processing speed. Two parallel test versions were used to minimize learning effects. SDMT scores were corrected for age, sex and educational level and transformed into *z*-scores, based on a normative sample of Dutch healthy controls (*n* = 407) [52].

Patients’ physical disability was measured with the Expanded Disability Status Scale (EDSS) questionnaire [53], the 9 Hole Peg Test (9-HPT) and the 25 Foot Walk Test (25-FWT) [54]. From these three, EDSS + progression was determined as a descriptive, binary, marker of physical disability progression [55].

To also take cognitive disability into account in this group of early RRMS subjects with relatively low EDSS scores, Multiple Sclerosis Functional Composite (MSFC) [54] scores were calculated from 9-HPT, 25-FWT and SDMT *z*-scores. For each of the three sections, results were considered impaired upon *z*-score ≤ -1.5. Over time, RRMS subjects were classified as MSFC decliner when MSFC scores were lower at year-2 compared to year-1, and as MSFC non-decliner when MSFC scores were equal or higher at year-2 compared to year-1.

### Statistics

HC vs RRMS comparisons for baseline demographics were performed by independent samples *t*-test, Mann–Whitney *U*-test, or Chi Square test, when appropriate. Differences in the RRMS group over time were analyzed with paired *t*-test, Wilcoxon Signed Ranks test, or McNemar test, when appropriate.

To analyze HC and RRMS differences over time in normalized brain volumes, cortical thickness, and *z*-scores for nDGM, CGM or WM, linear mixed models (LMM) with subject as random intercept were used, with fixed factors time, type (i.e. HC/RRMS) and time*type. When appropriate, LMM analysis was also performed to assess baseline differences between the two groups. Since application of an LMM requires constant variance in errors, *z*-scores for both WM and GM measures were used.

LMM analysis with subject as random intercept and type (i.e. HC/RRMS or MSFC non-decliner/decliner) as fixed factor was used for longitudinal analysis of DGM/CGM *z*-score relations with WM *z*-scores in the connected tracts. For the longitudinal relations, we analyzed both the effect of baseline WM *z*-scores on change in the connected GM *z*-scores (i.e. baseline WM to ΔGM), and the effect of baseline GM *z*-scores on change in WM *z*-scores in the connected tracts (i.e. baseline GM to ΔWM); both with type (HC/RRMS) or MSFC group (declining/non-declining) as covariates. When an interaction effects were present, the cohort was split based on type or MSFC group and LMM analysis were performed as described above. Analyses for DGM and CGM were performed separately.

Exploratory binary logistic regression was used to predict whether a subject would belong to the MSFC non-declining or MSFC declining group over time by looking at baseline WM or baseline GM values only; as well as combining these measures with demographics such as sex, age, education, treatment type and EDSS at baseline. Statistical analyses were performed using SPSS26 (IBM SPSS, Chicago, USA) and *p*-values were considered statistically relevant upon *p* ≤ 0.05. Since LMM analysis was performed multiple times (i.e. for every GM or WM measure as possible outcome separately), *p*-values were considered statistically significant upon *p* ≤ 0.01.

## Results

### Demographics

In total, 40 subjects with early RRMS and 15 age-and-sex-matched HCs were included in the study. A total of 11 subjects did not complete both year-1 and year-2 measurements and were, therefore, excluded from analyses in the current study (two HC subjects [inability to undergo MRI examination] and nine RRMS subjects [*n* = 5 switch to second-line therapy; *n* = 1 switch to first-line therapy just prior to examination; *n* = 3 inability to undergo MRI examination unrelated to RRMS]). This resulted in a total of 44 age-, sex-, and education-matched subjects (13 HC, 31 RRMS) with full data available for analysis, for whom demographics are depicted in Table 1. Follow-up time for RRMS and HC subjects was similar (1.00 ± 0.10 years and 0.99 ± 0.10 years, respectively).

**Table 1:** Demographics for HC and RRMS subjects; and RRMS clinical characteristics over time

Baseline demographics	HC (*n* = 13)	RRMS (*n* = 31)
**Age** in years, mean ± SD	37.5 ± 12.8	37.3 ± 7.5
**Sex**, m/f (% m)	4/9 (31)	7/24 (23)
**Education level**^a^, median (IQR)	6 (5–7)	6 (5–7)
**Follow-up time** in years, mean ± SD	0.99 ± 0.10	1.00 ± 0.10
**Disease duration** in years, mean ± SD	–	3.5 ± 1.4
**ARR**		
median (range)	–	0 (0–2)
≥ 1 relapse in previous year, *n* (%)		9 (29)
**Treatment**^b^, *n* (%)		
none	-	10 (32)
interferon		4 (13)
glatiramer acetate		5 (16)
dimethyl fumarate		11 (36)
teriflunomide		1 (3)
**Treatment duration** in years, mean ± SD	–	2.9 ± 1.1

Clinical characteristics	RRMS	RRMS
	year-1	year-2
**EDSS**		
median (range)	3.0 (0–5.0)	3.0 (0–6.0)
EDSS + progression, *n* (%)	–	6 (19)
**9-HPT**		
in seconds, mean ± SD	19.66 ± 2.95	19.44 ± 3.22
*z*-score, mean ± SD	–	0.108 ± 1.09
**25-FWT**		
in seconds, mean ± SD	4.40 ± 1.25	4.90 ± 2.19
*z*-score, mean ± SD	–	– 0.412 ± 1.785
**SDMT**		
*n* correct, mean ± SD	65 ± 14	66 ± 15
*z*-score, mean ± SD	– 0.387 ± 1.284	– 0.157 ± 1.289
**MSFC score**		
mean ± SD	– 0.129 ± 0.696	– 0.153 ± 0.880
≥ 1 impaired section, *n* (%)	10 (32)	7 (23)
MSFC decline over time, *n* (%)	n.a	15 (48)

*SD* standard deviation, *IQR* interquartile range; *ARR* annualized relapse rate, *EDSS* expanded disability status scale, *9-HPT* 9-hole peg test, *25-FWT* 25-foot walk test, *SDMT* symbol digit modalities test, *MSFC* multiple sclerosis functional composite

Statistics: *p < 0.05. *z*-scores from 9-HPT and 25-FWT were calculated from year-1 data, and are therefore not depicted at year-1 since they will, by default, have a mean of 0 and a SD of 1

^a^Education level according to Verhage scale (0–7)

^b^Treatment group interferon consists of interferon beta-1a (Avonex®, Rebif®), beta-1b (Betaferon®) and peginterferonbeta

RRMS subjects had an average disease duration of 3.5 ± 1.4 years at baseline measurements with median EDSS 3.0 (range 0.0–5.0). A total of 6 subjects (19%) showed EDSS + progression during follow-up. 9-HPT and SDMT scores did not significantly differ from year-1 to year-2, but worsening of 25-FWT results was seen (*Z* = – 2.930, *p* = 0.003). A total of ten subjects (32%) had at least one impaired MSFC section (i.e. 9-HPT, 25-FWT and/or SDMT) at year-1, and seven subjects (23%) at year-2. Total MSFC score did not change significantly over time, but a total of *n* = 15 subjects showed an overall decline in MSFC score from year-1 to year-2 (MSFC decliners), whereas *n* = 16 did not (MSFC non-decliners). No significant differences in age, sex, education, disease duration, treatment time/type, or number of relapses in the previous year were found between the MSFC decliners and non-decliners.

### MRI characteristics

MWI data were not available for 2 of 13 HC subjects and 7 of 31 RRMS subjects at year-1 (MSFC non-decliners *n* = 2 and MSFC decliners *n* = 5, respectively), and for 1 RRMS subject (MSFC non-decliner) at year-2. QSM data were not available for one RRMS subject (MSFC decliner) at year-1, and for one RRMS subject (MSFC non-decliner) at year-2.

#### HC vs RRMS subjects

MRI characteristics for HC and RRMS subjects over time are shown in Table 2. Larger atrophy rates in RRMS compared to HC subjects were found for normalized WB, WM and DGM volumes (*p* = 0.024, *p* = 0.018 and *p* = 0.026, respectively). RRMS subjects showed generally lower *z*-scores of DGM volume, CGM thickness, and measures of WM integrity (e.g. lower FA, higher MD; or more specifically lower NDI as axonal damage marker, and lower MWF as myelin damage marker), but no significant differences were found between HC and RRMS, neither cross-sectionally nor longitudinally.

**Table 2:** MRI characteristics of HC and RRMS subjects over time

	HC (*n* = 13)		RRMS (*n* = 31)		Statistics Time * Type interaction
	Year-1	Year-2	Year-1	Year-2	
**WB volume**					
Unnormalized (ml), mean ± SD	1134 ± 122	1123 ± 129	1071 ± 89	1066 ± 93	*n.a*
Normalized (%ICV), mean ± SD	70.4 ± 2.1	70.8 ± 2.2	70.0 ± 2.0	69.8 ± 2.3	*F*(1,42) = 5.473, *p* = 0.024 *
**WM volume**					
Unnormalized (ml), mean ± SD	419 ± 59	415 ± 61	393 ± 39	392 ± 42	*n.a*
Normalized (%ICV), mean ± SD	26.0 ± 1.1	26.1 ± 1.0	25.7 ± 1.2	25.6 ± 1.4	*F*(1,42) = 6.093, *p* = 0.018 *
**GM volume**					
Unnormalized (ml), mean ± SD	679 ± 64	673 ± 68	644 ± 52	641 ± 53	*n.a*
Normalized (%ICV), mean ± SD	42.2 ± 2.0	42.4 ± 2.1	42.1 ± 4.4	42.0 ± 1.6	*F*(1,42) = 3.589, *p* = 0.065
**DGM volume**^a^					*n.a*
Unnormalized (ml), mean ± SD	44.13 ± 3.08	44.16 ± 3.10	41.44 ± 3.68	41.25 ± 3.70	*F*(1,42) = 5.341, *p* = 0.026 *
Normalized (%ICV), mean ± SD	2.75 ± 0.19	2.76 ± 0.19	2.72 ± 0.17	2.70 ± 0.17	*B* = – 0.061, *p* = 0.571, CI [– 0.271, 0.149]
*z*-score, mean ± SD	0.145 ± 1.078	0.158 ± 1.067	– 0.040 ± 0.947	– 0.087 ± 0.972	*n.a*
**CGM thickness**^b^					
mm, mean ± SD	2.58 ± 0.10	2.57 ± 0.11	2.55 ± 0.10	2.54 ± 0.10	*F*(1,86) = 0.551, *p* = 0.460
*z*-score, mean ± SD	0.187 ± 1.022	0.099 ± 1.036	– 0.038 ± 0.967	– 0.083 ± 0.986	*B* = 0.043, *p* = 0.334, CI [– 0.044, 0.129]
**WM ***z*-**scores in DGM tracts**^c^					
WM-Diffusion	0.442 ± 0.829	0.105 ± 1.096	– 0.050 ± 0.867	– 0.180 ± 0.872	*B* = 0.207, *p* = 0.023, CI [0.028, 0.387] *
WM-Extended^d^	0.384 ± 0.688	0.177 ± 0.898	– 0.138 ± 0.839	– 0.169 ± 0.772	*B* = 0.290,* p* = 0.001, CI [0.114, 0.467] **
FA	0.504 ± 1.028	0.123 ± 1.094	– 0.013 ± 0.941	– 0.250 ± 0.911	*B* = 0.144, *p* = 0.131, CI [–0.043, 0.332]
MD	– 0.362 ± 0.817	– 0.014 ± 1.207	0.090 ± 0.952	0.105 ± 0.980	*B* = – 0.234, *p* = 0.022, CI [–0.452, –0.035] *
AD	– 0.006 ± 0.960	– 0.079 ± 1.127	0.132 ± 0.982	–0.096 ± 0.961	*B* = – 0.155, *p* = 0.183, CI [– 0.382, 0.073]
RD	– 0.462 ± 0.879	– 0.088 ± 1.224	0.046 ± 0.927	0.184 ± 0.946	*B* = – 0.235, *p* = 0.020, CI [– 0.433, –0.037] *
NDI	0.434 ± 0.82	0.221 ± 0.962	– 0.150 ± 0.985	– 0.125 ± 1.006	*B* = 0.238, *p* = 0.013, CI [0.051, 0.425] *
ODI	– 0.336 ± 0.982	0.032 ± 1.043	– 0.124 ± 0.965	0.251 ± 0.958	*B* = 0.007, *p* = 0.947, CI [– 0.211, 0.226]
MWF^d^	0.514 ± 0.592	0.347 ± 0.616	– 0.250 ± 1.424	– 0.139 ± 0.679	*B* = 0.342, *p* = 0.004, CI [0.108, 0.577] **
QSM^e^	0.053 ± 1.019	0.041 ± 0.943	– 0.052 ± 0.952	0.011 ± 1.057	*B* = 0.050, *p* = 0.706, CI [– 0.209, 0.309]
**WM ***z*-**scores in CGM tracts**^c^					
WM-Diffusion	0.320 ± 0.877	0.045 ± 1.002	0.033 ± 0.807	– 0.186 ± 0.919	*B* = 0.055, *p* = 0.136, CI [– 0.017, 0.128]
WM-Extended^d^	0.250 ± 0.788	0.123 ± 0.850	– 0.079 ± 0.743	– 0.189 ± 0.819	*B* = 0.122, *p* < 0.001, CI [0.050, 0.195] ***
FA	0.408 ± 1.001	– 0.068 ± 1.027	0.143 ± 0.940	– 0.286 ± 0.952	*B* = 0.048, *p* = 0.300, CI [– 0.043, 0.138]
MD	– 0.228 ± 0.971	0.154 ± 1.131	0.073 ± 0.880	0.087 ± 1.037	*B* = – 0.060, *p* = 0.132, CI [–0.139, 0.018]
AD	0.022 ± 1.045	– 0.264 ± 1.072	0.212 ± 0.928	– 0.111 ± 0.967	*B* = – 0.037, *p* = 0.425, CI [– 0.128, 0.054]
RD	– 0.323 ± 0.970	– 0.049 ± 1.143	– 0.030 ± 0.873	0.186 ± 1.020	*B* = – 0.058, *p* = 0.161, CI [– 0.139, 0.023]
NDI	0.293 ± 0.952	0.205 ± 0.970	– 0.073 ± 0.977	– 0.136 ± 1.009	*B* = 0.025, *p* = 0.469, CI [– 0.042, 0.092]
ODI	– 0.261 ± 1.014	0.368 ± 1.101	– 0.311 ± 0.879	0.266 ± 0.927	*B* = – 0.052, *p* = 0.293, CI [– 0.149, 0.045]
MWF^d^	0.495 ± 0.810	0.274 ± 0.792	– 0.171 ± 1.245	– 0.164 ± 0.813	*B* = 0.312, *p* < 0.001, CI [0.212, 0.412] ***
QSM^e^	0.154 ± 1.024	0.211 ± 0.938	– 0.118 ± 0.982	– 0.040 ± 1.004	*B* = 0.010, *p* = 0.852, CI [– 0.096, 0.116]

Bold values in statistics column are significant

*SD* standard deviation, *%ICV* percentage of total intra cranial volume, *B* estimate, *CI* 95% confidence interval

*p < 0.05; **p < 0.01; ***p < 0.001. Time*Type interaction calculated with HC as reference

^a^DGM volume is the total of the 10 structures (i.e. excluding left and right accumbens and amygdala)

^b^CGM thickness is calculated from the mean left and mean right cortical thickness output from FreeSurfer

^c^WM measures are depicted as a mean *z*-score over all DGM c.q. CGM tracts, respectively, and statistics calculated with linear mixed models to correct for the amount of tracts

^d^MWF was available for 11/13 HCs and 24/31 RRMS subjects at year-1, and for 13/13 HC and 30/31 RRMS subjects at year-2

^e^QSM was available for 13/13 HC and 30/31 RRMS subjects at year-1, and for 13/13 HC and 30/31 RRMS subjects at year-2

In both DGM- and CGM-seeded tracts, WM-Extended *z*-scores decreased less over time in RRMS compared to HC subjects (DGM: *B* = 0.290, SE = 0.090, *p* < 0.001; CGM:* B* = 0.122, SE = 0.037, *p* < 0.001), but the WM-Extended *z*-scores were also already lower at year-1 in RRMS compared to HC subjects (DGM: *B* = 0.464, SE = 0.200, *p* = 0.024; CGM: *B* = 0.358, SE = 0.190, *p* = 0.066), although not reaching significance.

#### MSFC non-declining vs MSFC declining subjects

Comparing the RRMS subjects based on their declining or non-declining MSFC score over time (Table 3), we found significantly higher atrophy rates for normalized WB, WM, GM and DGM volumes (*p* = 0.013, *p* = 0.029, *p* = 0.022 and *p* = 0.021, respectively) in the MSFC declining group compared to non-declining group. CGM thickness as well as CGM *z*-score also decreased faster over time in MSFC declining compared to non-declining subjects (*p* = 0.010 and *p* = 0.019, respectively).

**Table 3:** MRI characteristics of RRMS subjects with non-declining or declining MSFC score over time

	MSFC non-declining (*n* = 16)		MSFC declining (*n* = 15)		Statistics Time*Type interaction
	Year-1	year-2	Year-1	Year-2	
**WB volume**					
Unnormalized (ml), mean ± SD	1073 ± 93	1075 ± 96	1064 ± 94	1058 ± 92	*n.a*
Normalized (%ICV), mean ± SD	70.3 ± 2.0	70.4 ± 2.0	69.6 ± 2.1	69.2 ± 2.4	*F*(1,29) = 7.029, *p* = 0.013*
**WM volume**					
Unnormalized (ml), mean ± SD	392 ± 44	393 ± 45	392 ± 40	390 ± 39	*n.a*
Normalized (%ICV), mean ± SD	25.6 ± 1.2	25.7 ± 1.3	25.6 ± 1.4	25.5 ± 1.5	*F*(1,29) = 5.266, *p* = 0.029*
**GM volume**					
Unnormalized (ml), mean ± SD	648 ± 49	649 ± 51	638 ± 58	634 ± 56	*n.a*
Normalized (%ICV), mean ± SD	42.5 ± 1.4	42.5 ± 1.4	41.7 ± 1.5	41.4 ± 1.6	*F*(1,29) = 5.895, *p* = 0.022*
**DGM volume**^**a**^					
Unnormalized (ml), mean ± SD	42.12 ± 3.67	42.05 ± 3.71	40.71 ± 3.68	40.41 ± 3.62	*n.a*
Normalized (%ICV), mean ± SD	2.76 ± 0.17	2.76 ± 0.17	2.67 ± 0.16	2.65 ± 0.16	*F*(1,29) = 5.470, *p* = 0.026*
*z*-score, mean ± SD	0.164 ± 0.911	0.152 ± 0.918	– 0.257 ± 0.940	– 0.342 ± 0.967	*B* = 0.074, *p* = 0.511, CI [– 0.147, 0.296]
**CGM thickness**^**b**^					
mm, mean ± SD	2.56 ± 0.09	2.56 ± 0.09	2.53 ± 0.10	2.52 ± 0.10	*F*(1,60) = 7.165, *p* = 0.010**
*z*-score, mean ± SD	0.024 ± 0.949	0.033 ± 0.959	– 0.104 ± 0.983	– 0.207 ± 0.999	*B* = 0.113,* p* = 0.019, CI [0.018, 0.207]*
**WM** ***z*-scores in DGM tracts**^**c**^					
WM-Diffusion	0.045 ± 0.949	0.030 ± 0.870	– 0.151 ± 0.759	– 0.404 ± 0.820	*B* = 0.238,* p* = 0.009, CI [0.061, 0.415]**
WM-Extended^d^	– 0.115 ± 0.915	0.032 ± 0.751	– 0.171 ± 0.722	– 0.369 ± 0.742	*B* = 0.259,* p* = 0.006, CI [0.074, 0.444]**
FA	0.033 ± 0.992	– 0.110 ± 0.963	– 0.062 ± 0.883	– 0.398 ± 0.830	*B* = 0.194,* p* = 0.043, CI [0.006, 0.382]*
MD	– 0.046 ± 1.037	– 0.144 ± 0.920	0.235 ± 0.831	0.371 ± 0.974	*B* = – 0.235,* p* = 0.026, CI [– 0.442, – 0.028]*
AD	0.004 ± 1.009	– 0.262 ± 0.892	0.269 ± 0.936	0.080 ± 1.002	*B* = – 0.077,* p* = 0.517, CI [– 0.310, 0.156]
RD	– 0.057 ± 1.012	– 0.056 ± 0.926	0.156 ± 0.815	0.441 ± 0.903	*B* = – 0.285,* p* = 0.004, CI [– 0.480, – 0.089]**
NDI	– 0.020 ± 0.996	0.076 ± 0.951	– 0.289 ± 0.956	– 0.339 ± 1.022	*B* = 0.146,* p* = 0.132, CI [– 0.044, 0.337]
ODI	– 0.129 ± 0.966	0.260 ± 0.915	– 0.118 ± 0.967	0.242 ± 1.004	*B* = 0.030,* p* = 0.800, CI [– 0.201, 0.261]
MWF^d^	– 0.373 ± 1.711	0.018 ± 0.607	– 0.079 ± 0.856	– 0.296 ± 0.713	*B* = 0.602,* p* < 0.001, CI [0.307, 0.898]***
QSM^e^	0.045 ± 0.990	0.105 ± 1.062	– 0.163 ± 0.896	– 0.082 ± 1.047	*B* = 0.005,* p* = 0.973, CI [– 0.283, 0.293]
**WM** ***z*-scores in CGM tracts**^**c**^					
WM-Diffusion	0.185 ± 0.781	0.055 ± 0.788	– 0.128 ± 0.803	– 0.444 ± 0.977	*B* = 0.186,* p* < 0.001, CI [0.111, 0.261]***
WM-Extended^d^	0.010 ± 0.728	0.027 ± 0.698	– 0.204 ± 0.747	– 0.404 ± 0.874	*B* = 0.176,* p* < 0.001, CI [0.098, 0.254]***
FA	0.248 ± 0.952	– 0.128 ± 0.922	0.030 ± 0.913	– 0.453 ± 0.955	*B* = 0.107,* p* = 0.030, CI [0.011, 0.203]*
MD	– 0.112 ± 0.845	– 0.206 ± 0.850	0.270 ± 0.874	0.399 ± 1.1123	*B* = – 0.223,* p* < 0.001, CI [– 0.304, – 0.143]***
AD	0.065 ± 0.938	– 0.314 ± 0.894	0.370 ± 0.892	0.106 ± 0.995	*B* = – 0.116,* p* = 0.019, CI [– 0.213, – 0.019]*
RD	– 0.194 ± 0.842	– 0.088 ± 0.849	0.145 ± 0.871	0.478 ± 1.104	*B* = – 0.227,* p* < 0.001, CI [– 0.310, – 0.144]***
NDI	0.084 ± 0.949	0.056 ± 0.958	– 0.240 ± 0.979	– 0.341 ± 1.022	*B* = 0.073,* p* = 0.047, CI [0.001, 0.145]
ODI	– 0.332 ± 0.906	0.229 ± 0.916	– 0.288 ± 0.848	0.306 ± 0.938	*B* = – 0.033,* p* = 0.541, CI [– 0.137, 0.072]
MWF^d^	– 0.218 ± 1.433	0.020 ± 0.623	– 0.104 ± 0.916	– 0.347 ± 0.931	*B* = 0.486,* p* < 0.001, CI [0.368, 0.604]***
QSM^e^	– 0.114 ± 0.919	– 0.071 ± 0.908	– 0.123 ± 1.049	– 0.010 ± 1.091	*B* = – 0.050,* p* = 0.418, CI [– 0.170, 0.071]

Bold values in statistics column are significant

*SD* standard deviation, *%ICV* percentage of total intra cranial volume, *B* estimate, *CI* 95% confidence interval

*p < 0.05; **p < 0.01; ***p < 0.001. Time*Type interaction calculated with non-declining MSFC as reference

^a^DGM volume is the total of the 10 structures (i.e. excluding left and right accumbens and amygdala)

^b^CGM thickness is calculated from the mean left and mean right cortical thickness output from FreeSurfer

^c^WM measures are depicted as a mean *z*-score over all DGM c.q. CGM tracts, respectively, and statistics calculated with linear mixed models to correct for the amount of tracts

^d^MWF was available for 14/16 MSFC non-declining subjects and 10/15 MSFC declining subjects at year-1, and for 15/16 MSFC non-declining subjects and 15/15 MSFC declining subjects at year-2

^e^QSM was available for 16/16 MSFC non-declining and 14/15 MSFC declining subjects at year-1, and for 15/16 MSFC non-declining and 15/15 MSFC declining subjects at year-2

In both DGM and CGM tracts, several WM *z*-scores declined faster in MSFC declining compared to non-declining subjects (e.g. WM-Extended: *B* = 0.259, SE = 0.094, *p* = 0.006 for DGM, and *B* = 0.176, SE = 0.040, *p* < 0.001 for CGM, respectively). Interestingly, this was highly significant for changes in myelin-related measures (MWF and RD), but not for the axonal marker NDI. For the CGM, but not DGM, overall WM integrity was significantly lower at both time points in MSFC declining subjects compared to MSFC non-declining subjects (e.g. WM-Extended: *B* = – 0.436, SE = 0.204, *p* = 0.040).

### Effect of baseline WM integrity on change in connected GM over time

The relation between baseline WM integrity and changes in DGM volume and CGM thickness over time is shown in Table 4. Table 4A shows significant relations between baseline WM-Diffusion, WM-Extended, FA and RD *z*-scores and the change in CGM thickness in the entire cohort (e.g. WM-Extended: *B* = – 0.055, SE = 0.019, *p* = 0.005). This relationship between baseline WM and CGM thickness change was stronger in RRMS compared to HC subjects for WM-Diffusion, WM-Extended, MD, RD and NDI (e.g. WM-Diffusion*Type: *B* = 0.088, SE = 0.025, *p* < 0.001), thus including both axonal and myelin markers. When the MSFC non-declining group was compared with the MSFC declining group, similar effects were observed as when comparing HC with RRMS (Table 4B): also here the baseline WM *z*-scores had a WM*Type relationship in CGM (e.g. WM-Extended*MSFC type: *B* = 0.120, SE = 0.031, *p* = 0.001), where the relationship between WM damage and CGM thickness change was stronger in MSFC decliners than in non-decliners, and involved both markers of myelin damage in the WM (FA, RD) and the marker of axonal damage (NDI). No significant relations between WM *z*-scores and change in DGM volumes over time were seen, neither over the entire cohort, nor for the MSFC groups.

**Table 4:** Effect of baseline WM integrity *z*-score on change in DGM volume *z*-score and CGM thickness *z*-score over time; compared in HC/RRMS (A), MSFC non-declining/declining (B), and split per group (C; CGM only)

	ΔDGM			ΔCGM		
	WM	Type	WM*TYPE	WM	Type	WM*TYPE
**A. HC vs MS**						
WM-Diffusion						
*B*, *p*	0.026, 0.201	– 0.049, 0.076	– 0.014, 0.551	– 0.051, 0.002**	0.026, 0.622	0.073, < 0.001***
95% CI	[– 0.014, 0.066]	[– 0.102, 0.005]	[– 0.061, 0.033]	**[– 0.083, – 0.018]**	[– 0.079, 0.131]	**[0.033, 0.113]**
WM-Extended^a^						
*B*, *p*	0.006, 0.789	– 0.042, 0.099	– 0.004, 0.897	– 0.055, 0.005**	0.024, 0.697	0.088, < 0.001***
95% CI	[– 0.041, 0.054]	[– 0.093, 0.008]	[– 0.058, 0.051]	**[– 0.093, – 0.017]**	[– 0.101, 0.149]	**[0.040, 0.136]**
FA						
*B*, *p*	0.031, 0.059	– 0.045, 0.097	– 0.023, 0.254	– 0.038, 0.003**	0.027, 0.608	0.038, 0.015
95% CI	[– 0.001, 0.063]	[– 0.099, 0.008]	[– 0.063, 0.017]	**[– 0.064, – 0.013]**	[– 0.079, 0.133]	[0.008, 0.069]
MD						
*B*, *p*	– 0.003, 0.885	– 0.059, 0.028	– 0.007, 0.766	0.036, 0.024	0.037, 0.479	– 0.074, < 0.001***
95% CI	[– 0.041, 0.035]	[– 0.111, – 0.006]	[– 0.051, 0.037]	[0.005, 0.068]	[– 0.068, 0.142]	**[– 0.112, – 0.035]**
AD						
*B*, *p*	0.035, 0.028	– 0.060, 0.017	– 0.040, 0.035	– 0.004, 0.769	0.048, 0.363	– 0.022, 0.155
95% CI	[0.004, 0.065]	[– 0.109, – 0.011]	[– 0.076, – 0.003]	[– 0.030, 0.022]	[– 0.058, 0.154]	[– 0.053, 0.009]
RD						
*B*, *p*	– 0.024, 0.209	– 0.049, 0.073	0.012, 0.598	0.041, 0.007**	0.029, 0.583	– 0.062, < 0.001***
95% CI	[– 0.062, 0.014]	[– 0.103, 0.005]	[– 0.032, 0.056]	**[0.011, 0.071]**	[– 0.076, 0.134]	**[– 0.098, – 0.025]**
NDI						
*B*, *p*	– 0.008, 0.659	– 0.064, 0.021	0.007, 0.720	– 0.039, 0.024	0.034, 0.516	0.073, < 0.001***
95% CI	[– 0.042, 0.027]	[– 0.118, – 0.010]	[– 0.034, 0.048]	[– 0.073, – 0.005]	[– 0.070, 0.138]	**[0.033, 0.114]**
ODI						
*B*, *p*	– 0.040, 0.012	– 0.048, 0.066	0.033, 0.080	0.021, 0.088	0.042, 0.434	– 0.006, 0.672
95% CI	[– 0.071, – 0.009]	[– 0.100, 0.003]	[– 0.004, 0.070]	[– 0.003, 0.046]	[– 0.065, 0.149]	[– 0.036, 0.023]
MWF^a^						
*B*, *p*	– 0.031, 0.254	– 0.061, 0.026	0.032, 0.268	– 0.015, 0.393	0.032, 0.610	0.041, 0.045
95% CI	[– 0.086, 0.023]	[– 0.115, – 0.008]	[– 0.025, 0.089]	[– 0.049, 0.019]	[– 0.095, 0.160]	[0.001, 0.081]
QSM^b^						
*B*, *p*	0.009, 0.536	– 0.059, 0.028	0.008, 0.625	– 0.005, 0.659	0.039, 0.470	0.010, 0.461
95% CI	[– 0.019, 0.037]	[– 0.111, – 0.007]	[– 0.026, 0.043]	[– 0.029, 0.018]	[– 0.069, 0.147]	[– 0.017, 0.038]
**B. MSFC non-declining vs MSFC declining**						
WM-Diffusion						
*B*, *p*	– 0.003, 0.850	– 0.070, 0.018	0.029, 0.266	– 0.022, 0.188	– 0.109, 0.0510	0.083, < 0.001***
95% CI	[– 0.036, 0.029]	[– 0.127, – 0.013]	[– 0.023, 0.081]	[– 0.056, 0.011]	[– 0.219, 0.000]	**[0.037, 0.129]**
WM-Extended^a^						
*B*, *p*	– 0.004, 0.800	– 0.049, 0.079	0.019, 0.521	– 0.021, 0.306	– 0.090, 0.200	0.120, < 0.001***
95% CI	[– 0.038, 0.029]	[– 0.105, 0.006]	[– 0.041, 0.079]	[– 0.061, 0.019]	[– 0.232, 0.051]	**[0.059, 0.180]**
FA						
*B*, *p*	– 0.003, 0.874	– 0.073, 0.017	0.022, 0.369	– 0.026, 0.029	– 0.120, 0.034	0.056, 0.001**
95% CI	[– 0.034, 0.029]	[– 0.132, – 0.014]	[– 0.026, 0.069]	[– 0.049, 0.003]	[– 0.230, – 0.010]	**[0.021, 0.090]**
MD						
*B*, *p*	0.003, 0.834	– 0.069, 0.016	– 0.023, 0.316	– 0.009, 0.587	– 0.096, 0.084	– 0.049, 0.025
95% CI	[– 0.026, 0.032]	[– 0.125, – 0.014]	[– 0.069, 0.023]	[– 0.041, 0.023]	[– 0.206, 0.014]	[– 0.093, – 0.006]
AD						
*B*, *p*	0.002, 0.912	– 0.073, 0.013	– 0.006, 0.777	– 0.026, 0.025	– 0.105, 0.060	0.002, 0.930
95% CI	[– 0.026, 0.029]	[– 0.129, – 0.016]	[– 0.047, 0.035]	[– 0.049, – 0.003]	[– 0.216, 0.005]	[– 0.032, 0.035]
RD						
*B*, *p*	0.002, 0.894	– 0.070, 0.017	– 0.028, 0.244	0.020, 0.205	– 0.109, 0.051	– 0.074, < 0.001***
95% CI	[– 0.028, 0.032]	[– 0.127, – 0.013]	[– 0.077, 0.020]	[– 0.011, 0.051]	[– 0.219, 0.000]	**[– 0.116, – 0.031]**
NDI						
*B*, *p*	– 0.011, 0.453	– 0.072, 0.017	0.017, 0.460	0.003, 0.855	– 0.098, 0.085	0.059, 0.010**
95% CI	[– 0.041, 0.018]	[– 0.131, – 0.014]	[– 0.029, 0.63]	[– 0.029, 0.035]	[– 0.210, 0.014]	**[0.014, 0.104]**
ODI						
*B*, *p*	– 0.008, 0.578	– 0.074, 0.014	0.002, 0.938	0.025, 0.029	– 0.120, 0.034	– 0.021, 0.212
95% CI	[– 0.036, 0.020]	[– 0.131, – 0.016]	[– 0.040, 0.043]	[0.003, 0.047]	[– 0.231, – 0.009]	[– 0.053, 0.012]
MWF^a^						
*B*, *p*	– 0.008, 0.578	– 0.053, 0.057	– 0.001, 0.980	0.008, 0.547	– 0.106, 0.33	0.052, 0.025
95% CI	[– 0.036, 0.020]	[– 0.108, 0.002]	[– 0.047, 0.046]	[– 0.019, 0.035]	[– 0.246, 0.035]	[0.007, 0.097]
QSM^b^						
*B*, *p*	0.014, 0.261	– 0.072, 0.018	0.002, 0.907	– 0.014, 0.189	– 0.120, 0.037	0.037, 0.013
95% CI	[– 0.011, 0.040]	[– 0.131, – 0.014]	[– 0.038, 0.043]	[– 0.035, 0.007]	[– 0.233,– 0.008]	[0.008, 0.066]

	ΔCGM					
	HC (*n* = 13)	RRMS (*n* = 31)	MSFC non-declining (*n* = 16)	MSFC declining (*n* = 15)		
**C. Post hoc group split**						
WM-Diffusion						
*B*, *p*	– 0.051, 0.003	0.022, 0.057	– 0.022, 0.150	0.030, < 0.001***		
95% CI	[– 0.084, – 0.018]	[– 0.001, 0.046]	[– 0.053, 0.008]	**[0.026. 0.095]**		
WM-Extended^a^						
*B*, *p*	– 0.055, 0.005**	0.033, 0.030	– 0.021. 0.272	0.098. < 0.001***		
95% CI	**[– 0.093, – 0.017]**	[0.003, 0.063]	[– 0.059. 0.017]	**[0.050, 0.147]**		
FA						
*B*, *p*	– 0.038, 0.004**	0.000, 0.990	– 0.026, 0.016	0.030, 0.032		
95% CI	**[– 0.064, – 0.012]**	[– 0.017, 0.017]	[– 0.047, – 0.005]	[0.003, 0.058]		
MD						
*B*, *p*	0.036, 0.027	– 0.037, < 0.001***	– 0.009, 0.539	– 0.058, < 0.001***		
95% CI	[0.004, 0.069]	**[– 0.059, – 0.016]**	[– 0.039, 0.020]	**[– 0.090, – 0.027]**		
AD						
*B*, *p*	– 0.004, 0.770	– 0.026, 0.002**	– 0.027, 0.014	– 0.025, 0.065		
95% CI	[– 0.030, 0.023]	**[– 0.043, – 0.010]**	[– 0.048, – 0.005]	[– 0.052, 0.002]		
RD						
*B*, *p*	0.041, 0.008**	– 0.021, 0.057	0.020, 0.168	– 0.053, < 0.001***		
95% CI	**[0.011, 0.071]**	[– 0.042, 0.001]	[– 0.008, 0.048]	**[– 0.085, – 0.022]**		
NDI						
*B*, *p*	– 0.039, 0.027	0.034, 0.003**	0.002, 0.883	0.062, < 0.001****		
95% CI	[– 0.073, – 0.004]	**[0.012, 0.057]**	[– 0.027, 0.032]	**[0.028, 0.096]**		
ODI						
*B*, *p*	0.021, 0.094	0.015, 0.070	0.025, 0.016	0.004, 0.771		
95% CI	[– 0.004, 0.046]	[– 0.001, 0.031]	[0.005, 0.045]	[– 0.022, 0.030]		
MWF^a^						
*B*, *p*	– 0.015, 0.393	0.026, 0.017	0.008, 0.530	0.060, 0.003**		
95% CI	[– 0.049, 0.019]	[0.005, 0.048]	[– 0.017, 0.033]	**[0.021, 0.099]**		
QSM^b^						
*B*, *p*	– 0.005, 0.665	0.005, 0.492	– 0.014, 0.151	0.023, 0.042		
95% CI	[– 0.029, 0.019]	[– 0.009, 0.020]	[– 0.033, 0.005]	[0.001, 0.045]		

Bold values in statistics column are significant

*B* estimate, CI confidence interval

**p < 0.01; ***p < 0.001

^a^MWF was available for 11/13 HCs and 24/31 RRMS subjects (MSFC non-declining *n* = 15, MSFC declining *n* = 10) at year-1, and for 13/13 HC and 30/31 RRMS subjects (MSFC non-declining *n* = 15, MSFC declining *n* = 15) at year-2

^b^QSM was available for 13/13 HC and 30/31 RRMS subjects (MSFC non-declining *n* = 16, MSFC declining *n* = 14) at year-1, and for 13/13 HC and 30/31 RRMS subjects (MSFC non-declining *n* = 15, MSFC declining *n* = 15) at year-2

Post hoc analysis in HC and RRMS groups separately (Table 4C) showed that lower WM integrity related to a decrease in cortical thickness over time in RRMS subjects (e.g. WM-Extended: *B* = 0.033, SE = 0.015, *p* = 0.030), whereas HC subjects showed an opposite relationship (e.g. WM-Extended: *B* = – 0.055, SE = 0.019, *p* = 0.004). Post hoc analysis splitting in the two MSFC groups showed that the WM–GM relationship was only significant in subjects whose MSFC score declined over time (e.g. WM-Extended: *B* = 0.098, SE = 0.025, *p* < 0.001), and not in subjects whose MSFC did not (e.g. WM-Extended: *B* = – 0.021, SE = 0.019, *p* = 0.272).

### Effect of baseline GM on change in connected WM integrity over time

The relation between baseline DGM volume or CGM thickness and changes in WM integrity over time is shown in Table 5. Table 5A shows there were no significant relations between baseline GM *z*-scores and WM *z*-scores over time, except for a different association in HC and RRMS subjects of baseline DGM volume on AD change over time, which was also seen between the two MSFC groups (Table 5B). CGM thickness *z*-scores did not relate significantly to WM change over time.

**Table 5:** Effect of baseline DGM volume *z*-score or CGM thickness *z*-score on change in WM integrity *z*-score over time; compared in HC/RRMS (A), MSFC non-declining/declining (B), and split per group (C; DGM only)

	DGM			CGM		
	WM	Type	WM*TYPE	WM	Type	WM*TYPE
**A. HC vs MS**						
ΔWM-Diffusion						
*B*, *p*	0.009, 0.867	0.209, 0.201	– 0.007, 0.911	– 0.001, 0.965	0.055, 0.720	0.008, 0.760
95% CI	[– 0.095, 0.113]	[– 0.115, 0.533]	[– 0.136, 0.121]	[– 0.042, 0.040]	[– 0.253, 0.364]	[– 0.042, 0.057]
ΔWM-Extended^a^						
*B*, *p*	0.015, 0.791	0.306, 0.089	– 0.060, 0.389	– 0.010, 0.642	0.121, 0.465	0.025, 0.341
95% CI	[– 0.094, 0.123]	[– 0.049, 0.660]	[– 0.195, 0.076]	[– 0.051, 0.032]	[– 0.213, 0.456]	[– 0.026, 0.075]
ΔFA						
*B*, *p*	– 0.046, 0.330	0.139, 0.252	0.083, 0.160	0.011, 0.651	0.049, 0.663	– 0.020, 0.478
95% CI	[– 0.140, 0.047]	[– 0.103, 0.381]	[– 0.033, 0.198]	[– 0.036, 0.058]	[– 0.178, 0.277]	[– 0.076, 0.036]
ΔMD						
*B*, *p*	– 0.068, 0.286	– 0.251, 0.189	0.107, 0.174	0.010, 0.684	– 0.059, 0.745	– 0.031, 0.281
95% CI	[– 0.193, 0.057]	[– 0.631, 0.128]	[– 0.047, 0.261]	[– 0.038, 0.057]	[– 0.424, 0.306]	[– 0.088, 0.025]
ΔAD						
*B*, *p*	– 0.129, 0.041	– 0.169, 0.240	0.235, 0.003**	0.005, 0.841	– 0.037, 0.754	– 0.033, 0.287
95% CI	[– 0.254, – 0.005]	[– 0.455, 0.117]	**[0.081, 0.389]**	[– 0.046, 0.056]	[– 0.273, 0.200]	[– 0.094, 0.028]
ΔRD						
*B*, *p*	– 0.008, 0.888	– 0.237, 0.210	– 0.001, 0.987	0.003, 0.903	– 0.058, 0.753	– 0.013, 0.656
95% CI	[– 0.125, 0.108]	[– 0.611, 0.138]	[– 0.145, 0.143]	[– 0.044, 0.049]	[– 0.424, 0.309]	[– 0.068, 0.043]
ΔNDI						
*B*, *p*	0.018, 0.774	0.239, 0.068	– 0.044, 0.562	– 0.009, 0.670	0.024, 0.815	0.022, 0.356
95% CI	[– 0.103, 0.138]	[– 0.018, 0.497]	[– 0.192, 0.105]	[– 0.049, 0.031]	[– 0.180, 0.227]	[– 0.025, 0.070]
ΔODI						
*B*, *p*	0.087, 0.099	0.017, 0.850	– 0.150, 0.022	– 0.036, 0.1169	– 0.058, 0.474	0.053, 0.085
95% CI	[– 0.016, 0.190]	[– 0.167, 0.202]	[– 0.277, – 0.022]	[– 0.086, 0.015]	[– 0.221, 0.104]	[– 0.007, 0.114]
ΔMWF^a^						
*B*, *p*	0.028, 0.645	0.363, 0.407	– 0.075, 0.333	– 0.025, 0.465	0.308, 0.385	0.093, 0.026
95% CI	[– 0.093, 0.150]	[– 0.517, 1.243]	[– 0.228, 0.078]	[– 0.093, 0.042]	[– 0.405, 1.021]	[0.011, 0.176]
ΔQSM^b^						
*B*, *p*	0.068, 0.320	0.051, 0.593	– 0.066, 0.440	– 0.004, 0.914	0.009, 0.906	0.019, 0.643
95% CI	[– 0.067, 0.203]	[– 0.139, 0.241]	[– 0.235, 0.103]	[– 0.069, 0.062]	[– 0.148, 0.167]	[– 0.060, 0.097]
**B. MSFC non-declining vs MSFC declining**						
ΔWM-Diffusion						
*B*, *p*	0.035, 0.459	– 0.241, 0.100	– 0.070, 0.277	0.028, 0.110	– 0.0187, 0.148	– 0.043, 0.086
95% CI	[– 0.059, 0.129]	[– 531, 0.049]	[– 0.196, 0.056]	[– 0.006, 0.062]	[– 0.444, 0.070]	[– 0.092, 0.006]
ΔWM-Extended^a^						
*B*, *p*	– 0.017, 0.719	– 0.285, 0.114	– 0.074, 0.274	0.032, 0.071	– 0.172, 0.259	– 0.037, 0.156
95% CI	[– 0.111, 0.077]	[– 0.646, 0.075]	[– 0.207, 0.059]	[– 0.003, 0.066]	[– 0.479, 0.136]	[– 0.088, 0.014]
ΔFA						
*B*, *p*	0.040, 0.334	– 0.181, 0.104	– 0.016, 0.772	– 0.006, 0.782	– 0.108, 0.312	– 0.007, 0.812
95% CI	[– 0.041, 0.120]	[– 0.402, 0.040]	[– 0.125, 0.093]	[– 0.046, 0.035]	[– 0.324, 0.107]	[– 0.065, 0.051]
ΔMD						
*B*, *p*	– 0.025, 0.664	0.257, 0.150	0.127, 0.107	– 0.051, 0.011	0.223, 0.136	0.060, 0.035
95% CI	[– 0.139, 0.089]	[– 0.097, 0.610]	[– 0.027, 0.281]	[– 0.090, – 0.012]	[– 0.074, 0.521]	[0.004, 0.116]
ΔAD						
*B*, *p*	– 0.005, 0.940	0.131, 0.390	0.216, 0.010**	– 0.042, 0.057	0.114, 0.276	0.031, 0.334
95% CI	[– 0.125, 0.116]	[– 0.175, 0.437]	**[0.052, 0.380]**	[– 0.086, 0.001]	[– 0.096, 0.323]	[– 0.032, 0.093]
ΔRD						
*B*, *p*	– 0.042, 0.438	0.285, 0.087	0.068, 0.350	– 0.040, 0.042	0.228, 0.137	0.061, 0.028
95% CI	[– 0.147, 0.064]	[– 0.044, 0.613]	[– 0.075, 0.210]	[– 0.078, – 0.002]	[– 0.077, 0.534]	[0.007, 0.116]
ΔNDI						
*B*, *p*	– 0.012, 0.831	– 0.162, 0.191	– 0.039, 0.613	0.037, 0.034	– 0.073, 0.470	– 0.047, 0.056
95% CI	[– 0.125, 0.100]	[– 0.408, 0.085]	[– 0.193, 0.114]	[0.003, 0.071]	[– 0.278, 0.132]	[– 0.096, 0.001]
ΔODI						
*B*, *p*	– 0.023, 0.669	– 0.062, 0.548	– 0.089, 0.222	0.022, 0.343	0.034, 0.686	– 0.008, 0.812
95% CI	[– 0.127, 0.081]	[– 0.271, 0.147]	[– 0.231, 0.054]	[– 0.023, 0.066]	[– 0.138, 0.207]	[– 0.071, 0.056]
ΔMWF^a^						
*B*, *p*	– 0.004, 0.957	– 0.634, 0.284	– 0.098,0.323	0.082, 0.014	– 0.468, 0.314	– 0.031, 0.525
95% CI	[– 0.141, 0.134]	[– 1.835, 0.566]	[– 0.292, 0.097]	[0.016, 0.147]	[– 1.410, 0.475]	[– 0.129, 0.066]
ΔQSM^b^						
*B*, *p*	– 0.075, 0.296	– 0.006, 0.958	0.156, 0.130	0.030, 0.340	0.049, 0.549	– 0.033, 0.468
95% CI	[– 0.218, 0.067]	[– 0.219, 0.208]	[– 0.046, 0.358]	[– 0.032, 0.093]	[– 0.117, 0.215]	[– 0.121, 0.056]

	DGM			
	HC (*n* = 13)	RRMS (*n* = 31)	MSFC non-declining (*n* = 16)	MSFC declining (*n* = 15)
**C. Post hoc group split**				
ΔWM-Diffusion				
*B*, *p*	0.009, 0.900	0.001, 0.963	0.035, 0.493	– 0.034, 0.401
95% CI	[– 0.129, 0.147]	[– 0.061, 0.064]	[– 0.065, 0.134]	[– 0.113, 0.046]
ΔWM-Extended^a^				
*B*, *p*	0.017, 0.814	– 0.049, 0.143	– 0.022, 0.665	– 0.083, 0.059
95% CI	[– 0.127, 0.161]	[– 0.116, 0.017]	[– 0.121, 0.077]	[– 0.169, 0.003]
ΔFA				
*B*, *p*	– 0.045, 0.486	0.036, 0.198	0.040, 0.355	0.024, 0.511
95% CI	[– 0.174, 0.083]	[– 0.019, 0.090]	[– 0.045, 0.124]	[– 0.047, 0.094]
ΔMD				
*B*, *p*	– 0.068, 0.410	0.039, 0.314	– 0.024, 0.696	0.099, 0.050
95% CI	[– 0.230, 0.095]	[– 0.037, 0.116]	[– 0.142, 0.095]	[0.000, 0.198]
ΔAD				
*B*, *p*	– 0.130, 0.086	0.109, 0.009**	– 0.002, 0.977	0.203, < 0.001***
95% CI	[– 0.279, 0.019]	**[0.027, 0.190]**	[– 0.132, 0.128]	**[0.103, 0.302]**
ΔRD				
*B*, *p*	– 0.008, 0.919	– 0.010, 0.783	– 0.041, 0.468	0.025, 0.582
95% CI	[– 0.162, 0.146]	[– 0.081, 0.061]	[– 0.153, 0.071]	[– 0.065, 0.115]
ΔNDI				
*B*, *p*	0.018, 0.820	– 0.026, 0.497	– 0.011, 0.856	– 0.046, 0.338
95% CI	[– 0.135, 0.170]	[– 0.102, 0.050]	[– 0.133, 0.110]	[– 0.139, 0.048]
ΔODI				
*B*, *p*	0.087, 0.150	– 0.066, 0.066	– 0.023, 0.679	– 0.113, 0.017
95% CI	[– 0.032, 0.206]	[– 0.136, 0.004]	[– 0.133, 0.087]	[– 0.205, – 0.021]
ΔMWF^a^				
*B*, *p*	0.038, 0.493	– 0.050, 0.307	– 0.010, 0.898	– 0.104, 0.079
95% CI	[– 0.071, 0.147]	[– 0.148, 0.047]	[– 0.162, 0.142]	[– 0.220, 0.012]
ΔQSM^b^				
*B*, *p*	0.073, 0.28	0.001, 0.978	– 0.078, 0.216	0.091, 0.263
95% CI	[– 0.075, 0.221]	[– 0.097, 0.100]	[– 0.201, 0.046]	[– 0.070, 0.253]

Bold values in statistics column are significant

*B* estimate, CI confidence interval

**p < 0.01; ***p < 0.001

^a^MWF was available for 11/13 HCs and 24/31 RRMS subjects (MSFC non-declining *n* = 15, MSFC declining *n* = 10) at year-1, and for 13/13 HC and 30/31, RRMS subjects (MSFC non-declining *n* = 15, MSFC declining *n* = 15) at year-2

^b^QSM was available for 13/13 HC and 30/31 RRMS subjects (MSFC non-declining *n* = 16, MSFC declining *n* = 14) at year-1, and for 13/13 HC and 30/31 RRMS subjects (MSFC non-declining *n* = 15, MSFC declining *n* = 15) at year-2

Post hoc analysis in HC and RRMS groups separately (Table 5C) showed that in RRMS subjects, lower DGM volumes related to a decrease in AD in the connected tracts over time (*B* = 0.109, SE = 0.042, *p* = 0.009), an effect that was not found in HC subjects (*B* = – 0.130, SE = 0.075, *p* = 0.086). Furthermore, this was only seen in subjects whose MSFC would decline over time (*B* = 0.203, SE = 0.050, *p* < 0.001), and not in subjects whose MSFC would not decline over time (*B* = – 0.002, SE = 0.066, *p* = 0.977).

### Predicting MSFC decline based on baseline WM and GM parameters

Exploratory binary logistic regression to study the relation between baseline imaging parameters and the MSFC groups showed that WM-Extended *z*-scores were better predictors than WM-Diffusion *z*-scores (Nagelkerke R^2^ = 0.594 versus 0.362, respectively), and that either of the WM *z*-scores were better predictors than GM *z*-scores only (Nagelkerke *R*^2^ = 0.287) in a model including sex, age, education, treatment type and baseline EDSS score. A total of 78% of subjects could be classified correctly (MSFC-non-declining: 84%; MSFC-declining: 69%) in a model based on sex, age, education, treatment type, EDSS, GM *z*-score, WM-Diffusion *z*-score and WM-Extended *z*-score (Nagelkerke *R*^2^ = 0.611).

## Discussion

This longitudinal study in early RRMS explored the spatial and temporal relations between GM damage and WM integrity in the connected tracts using multimodal MRI. Our most important finding was that lower baseline WM integrity, as determined with DTI, NODDI and MWF, related to increasing atrophy of the connected cortical GM over time in subjects with RRMS, and especially for those who experienced increasing disability over the study period. In contrast, lower baseline cortical GM thickness or deep GM volume did not relate to WM integrity changes in the connected tracts over time.

These results suggest that in early RRMS, damage of the WM precedes atrophy of the cortical GM and that this relationship is clinically relevant since it was only found in subjects with worsening of their overall MSFC score over time. Cortical thinning as a result of preceding lower WM integrity in connected tracts has been suggested previously [56, 57], and we extend those findings by studying this longitudinally in early RRMS. Accelerated CGM atrophy has previously also been found upon increasing clinical progression independent of relapse activity [58], and our results confirm this already in the very early stages of the disease. For progressive MS, it was earlier found that WM damage preceded cortical GM damage [6], and a recent combined MRI-histopathological study showed that this WM-integrity-related cortical thinning could be attributed to GM axonal density loss, rather than myelin or microglia density loss within GM [59].

Our study adds important new insights, by demonstrating that in WM tracts, both axonal damage and myelin damage are related to subsequent cortical thinning. That myelin damage, as assessed here through MWF and RD, would be implicated, may not come as a surprise given the relations between demyelinating focal WM lesions and GM atrophy in MS described previously and reviewed systematically in [9]***.*** Nonetheless, the fact that quantitatively assessed myelin damage in whole WM tracts was related to subsequent thinning of the connected cortex in RRMS, is novel. While (higher) RD was highly significantly more strongly related to subsequent cortical thinning in RRMS than HC, (lower) MWF failed to reach significance. In the post hoc analyses in RRMS alone, both RD and MWF just failed to meet the significance threshold. However, in the most affected patient group, those with RRMS who exhibited MSFC decline during the study, both RD and MWF at baseline were highly significantly related to subsequent thinning of the connected cortex. Future studies, ideally in larger groups but maintaining the homogeneity of image acquisition adhered to here, should investigate the role of myelin damage in WM in the process of cortical thinning in RRMS in more detail.

Axonal damage is another important component of WM damage in MS. With NDI, this study included a quantitative marker of this damage. Lower NDI in WM tracts was significantly related to more subsequent thinning of the connected cortex in; whether assessing its effect in RRMS versus HC; in MSFC-declining versus non-declining RRMS; or in the post hoc analyses on RRMS separately and in the MSFC-declining subgroup. The recurring strength of this marker suggests an important role for WM axonal damage in the development of subsequent cortical thinning.

The finding that this process may already be ongoing in early RRMS may also inform therapeutic choices: treatments that would be developed to target primarily neurodegeneration may have limited effect, since atrophy may be secondary to WM damage. In the current study, we did not find any significant influences of WM integrity on atrophy of DGM in either HC or RRMS subjects; and also not in either of the two MSFC groups. Correlations between WM damage and connected DGM volumes have been shown in earlier studies, although these studies focused only on lesion volume and not on overall integrity of the entire tract [9, 60]. Studies that did look at overall integrity of the tract at baseline and its effect on atrophy focused mainly on the thalamus [7, 61, 62]. Interestingly, a cross-sectional study showed that connectivity of thalamocortical tracts related to cortical, but not thalamic, atrophy [63], which may be in line with our results where we did not find any relations between WM and DGM atrophy, but only with CGM atrophy. It is known that MS DGM segmentation has some methodological issues[64], and therefore we took some precautions: subjects’ brain volumes were measured at similar times of the day to limit diurnal volume fluctuations; specific longitudinal segmentation software optimized in subjects with MS was used[34]; amygdala and nucleus accumbens were excluded from analysis due to known difficulties in segmentation of these small structures[36]; and DGM volumes were normalized and converted into *z*-scores per structure to enable grouping of large and small DGM structures within one LMM. However, where our linear mixed model method was well suited for analysis of the CGM tracts (i.e. damage may occur in different tracts and different cortical areas between MS subjects), it may be less suitable for the analysis of DGM tracts, and analyses per DGM structure may be more informative. Due to the small sample size, we did not further investigate the separate DGM structures in the current study, but this remains of interest for future, larger, studies.

The fact that anterograde neurodegeneration from WM integrity loss was mainly found in subjects who showed clinical progression (i.e. MSFC decliners), further indicates the clinical relevance of these disease mechanisms. It should be noted that the distinction between decliner and non-decliner is only subtle, in the 1-year follow-up of this cohort of 31 RRMS subjects. However, although the groups only consisted of 15 declining and 16 non-declining RRMS subjects, exploratory regression analysis suggested importance of baseline WM integrity (including NDI and MWF) over baseline GM scores, on MSFC progression over time. Although implications for clinical progression are subtle and preliminary, future studies may benefit from including measures of the integrity of the tract over lesion volume only, and especially from the addition of NDI and MWF as important axonal and myelin markers, respectively, to standard DTI analyses.

This study has some limitations. First, our sample size was relatively small and for a few subjects, MWF and QSM values were not available; this was mitigated by using mixed models analyses that take all data-points into account. Although our study follow-up time was short, significant atrophy patterns as well as WM–GM relations could be recognized already early in the disease. Larger sample sizes and longer follow-up times might increase the power of these findings.

Furthermore, while it would be interesting to study the relation with GM atrophy separately for damage in WM lesions in connected tracts and for damage in NAWM in connected tracts, we have chosen not to pursue such analyses in the present work. Because of the small numbers of lesions per person in our study group of people with early MS, and because the anatomical locations of those lesions vary from patient to patient, a large number of WM tracts will not contain any lesion. The small amount of data will lead to a low statistical power regarding the relation between that lesion-bound damage on the one hand, and atrophy of the connected GM regions on the other. By contrast, by quantifying the tract-specific damage through the quantitative MR measures for the whole tract, regardless of whether lesions were present, we were able to include all tracts in all patients in our analyses. This allowed for a comprehensive analysis, while also limiting the number of statistical tests. Nonetheless, the extraction of weighted mean values from the whole WM tract may be less optimal for QSM, due to opposite effects on susceptibility of demyelination and iron deposition[15], and so for QSM separate analysis of lesions, perilesional WM and NAWM may be informative. For future studies, it may be interesting to also look at the microstructural measures in the DGM and CGM itself. In this way, we can investigate not only the effect of WM integrity on atrophy of the connected GM, but also on the neurobiological processes underlying this neurodegeneration, such as demyelination and axonal loss.

## Conclusion

Lower baseline WM integrity related to increasing cortical atrophy in RRMS subjects that show clinical progression over a 1-year follow-up. Baseline GM did not influence WM integrity changes over time. Collectively, this suggests that white matter tract damage drives cortical neurodegeneration in early RRMS.

## Data Availability

Anonymized data not published within this article will be made available by reasonable request from any qualified investigator.

## References

[1] Geurts JJ, Calabrese M, Fisher E, Rudick RA (2012). Measurement and clinical effect of grey matter pathology in multiple sclerosis. Lancet Neurol.

[2] Benedict RH, Ramasamy D, Munschauer F, Weinstock-Guttman B, Zivadinov R (2009). Memory impairment in multiple sclerosis: correlation with deep grey matter and mesial temporal atrophy. J Neurol Neurosurg Psychiatry.

[3] Zivadinov R, Bergsland N, Dolezal O, Hussein S, Seidl Z, Dwyer MG, Vaneckova M, Krasensky J, Potts JA, Kalincik T, Havrdova E, Horakova D (2013). Evolution of cortical and thalamus atrophy and disability progression in early relapsing-remitting MS during 5 years. AJNR Am J Neuroradiol.

[4] Uher T, Blahova-Dusankova J, Horakova D, Bergsland N, Tyblova M, Benedict RH, Kalincik T, Ramasamy DP, Seidl Z, Hagermeier J, Vaneckova M, Krasensky J, Havrdova E, Zivadinov R (2014). Longitudinal MRI and neuropsychological assessment of patients with clinically isolated syndrome. J Neurol.

[5] Steenwijk MD, Daams M, Pouwels PJ, Balk J, Tewarie PK, Geurts JJ, Barkhof F, Vrenken H (2015). Unraveling the relationship between regional gray matter atrophy and pathology in connected white matter tracts in long-standing multiple sclerosis. Hum Brain Mapp.

[6] Bodini B, Chard D, Altmann DR, Tozer D, Miller DH, Thompson AJ, Wheeler-Kingshott C, Ciccarelli O (2016). White and gray matter damage in primary progressive MS: the chicken or the egg?. Neurology.

[7] Kolasinski J, Stagg CJ, Chance SA, Deluca GC, Esiri MM, Chang EH, Palace JA, McNab JA, Jenkinson M, Miller KL, Johansen-Berg H (2012). A combined post-mortem magnetic resonance imaging and quantitative histological study of multiple sclerosis pathology. Brain.

[8] Jehna M, Langkammer C, Khalil M, Fuchs S, Reishofer G, Fazekas F, Ebner F, Enzinger C (2013). An exploratory study on the spatial relationship between regional cortical volume changes and white matter integrity in multiple sclerosis. Brain Connect.

[9] Lie IA, Weeda MM, Mattiesing RM, Mol MAE, Pouwels PJW, Barkhof F, Torkildsen O, Bo L, Myhr KM, Vrenken H (2022). Relationship between white matter lesions and gray matter atrophy in multiple sclerosis: a systematic review. Neurology.

[10] Jones DK, Knosche TR, Turner R (2013). White matter integrity, fiber count, and other fallacies: the do's and don'ts of diffusion MRI. Neuroimage.

[11] Zhang H, Schneider T, Wheeler-Kingshott CA, Alexander DC (2012). NODDI: practical in vivo neurite orientation dispersion and density imaging of the human brain. Neuroimage.

[12] Margoni M, Villani U, Silvestri E, Franciotta S, Anglani MG, Causin F, Rinaldi F, Perini P, Bertoldo A, Gallo P (2022). Quantification of normal-appearing white matter damage in early relapse-onset multiple sclerosis through neurite orientation dispersion and density imaging. Mult Scler Relat Disord.

[13] Alotaibi A, Podlasek A, AlTokhis A, Aldhebaib A, Dineen RA, Constantinescu CS (2021). Investigating microstructural changes in white matter in multiple sclerosis: a systematic review and meta-analysis of neurite orientation dispersion and density imaging. Brain Sci.

[14] Qian W, Khattar N, Cortina LE, Spencer RG, Bouhrara M (2020). Nonlinear associations of neurite density and myelin content with age revealed using multicomponent diffusion and relaxometry magnetic resonance imaging. Neuroimage.

[15] Zhang Y, Gauthier SA, Gupta A, Comunale J, Chia-Yi Chiang G, Zhou D, Chen W, Giambrone AE, Zhu W, Wang Y (2016). Longitudinal change in magnetic susceptibility of new enhanced multiple sclerosis (MS) lesions measured on serial quantitative susceptibility mapping (QSM). J Magn Reson Imaging.

[16] MacKay AL, Laule C (2016). Magnetic resonance of myelin water: an in vivo Marker for Myelin. Brain Plast.

[17] Polman CH, Reingold SC, Banwell B, Clanet M, Cohen JA, Filippi M, Fujihara K, Havrdova E, Hutchinson M, Kappos L, Lublin FD, Montalban X, O'Connor P, Sandberg-Wollheim M, Thompson AJ, Waubant E, Weinshenker B, Wolinsky JS (2011). Diagnostic criteria for multiple sclerosis: 2010 revisions to the McDonald criteria. Ann Neurol.

[18] De Stefano N, Matthews PM, Filippi M, Agosta F, De Luca M, Bartolozzi ML, Guidi L, Ghezzi A, Montanari E, Cifelli A, Federico A, Smith SM (2003). Evidence of early cortical atrophy in MS: relevance to white matter changes and disability. Neurology.

[19] Rao AB, Richert N, Howard T, Lewis BK, Bash CN, McFarland HF, Frank JA (2002). Methylprednisolone effect on brain volume and enhancing lesions in MS before and during IFNbeta-1b. Neurology.

[20] Weeda MM, Zywicki S, Brouwer I, Moraal B, Killestein J, Gallo P, Barkhof F, Pouwels PJW, Vrenken H (2022). Upper cervical cord atrophy is independent of cervical cord lesion volume in early multiple sclerosis: a two-year longitudinal study. Mult Scler Relat Disord.

[21] Wang Y, Ma X, Zhang Z, Dai E, Jeong HK, Xie B, Yuan C, Guo H (2018). A comparison of readout segmented EPI and interleaved EPI in high-resolution diffusion weighted imaging. Magn Reson Imaging.

[22] Popescu V, Battaglini M, Hoogstrate WS, Verfaillie SC, Sluimer IC, van Schijndel RA, van Dijk BW, Cover KS, Knol DL, Jenkinson M, Barkhof F, de Stefano N, Vrenken H, Group MS (2012). Optimizing parameter choice for FSL-Brain Extraction Tool (BET) on 3D T1 images in multiple sclerosis. Neuroimage.

[23] Smith SM, Zhang Y, Jenkinson M, Chen J, Matthews PM, Federico A, De Stefano N (2002). Accurate, robust, and automated longitudinal and cross-sectional brain change analysis. Neuroimage.

[24] Zheng W, Chee MW, Zagorodnov V (2009). Improvement of brain segmentation accuracy by optimizing non-uniformity correction using N3. Neuroimage.

[25] Valverde S, Salem M, Cabezas M, Pareto D, Vilanova JC, Ramio-Torrenta L, Rovira A, Salvi J, Oliver A, Llado X (2019). One-shot domain adaptation in multiple sclerosis lesion segmentation using convolutional neural networks. Neuroimage Clin.

[26] Valverde S, Cabezas M, Roura E, Gonzalez-Villa S, Pareto D, Vilanova JC, Ramio-Torrenta L, Rovira A, Oliver A, Llado X (2017). Improving automated multiple sclerosis lesion segmentation with a cascaded 3D convolutional neural network approach. Neuroimage.

[27] Weeda MM, Brouwer I, de Vos ML, de Vries MS, Barkhof F, Pouwels PJW, Vrenken H (2019). Comparing lesion segmentation methods in multiple sclerosis: Input from one manually delineated subject is sufficient for accurate lesion segmentation. Neuroimage Clin.

[28] Chard DT, Jackson JS, Miller DH, Wheeler-Kingshott CA (2010). Reducing the impact of white matter lesions on automated measures of brain gray and white matter volumes. J Magn Reson Imaging.

[29] Reuter M, Schmansky NJ, Rosas HD, Fischl B (2012). Within-subject template estimation for unbiased longitudinal image analysis. Neuroimage.

[30] Dale AM, Fischl B, Sereno MI (1999). Cortical surface-based analysis. I/ Segmentation and surface reconstruction. Neuroimage.

[31] Fischl B, Sereno MI, Dale AM (1999). Cortical surface-based analysis. II: inflation, flattening, and a surface-based coordinate system. Neuroimage.

[32] Guo C, Ferreira D, Fink K, Westman E, Granberg T (2019). Repeatability and reproducibility of FreeSurfer, FSL-SIENAX and SPM brain volumetric measurements and the effect of lesion filling in multiple sclerosis. Eur Radiol.

[33] Klasson N, Olsson E, Rudemo M, Eckerstrom C, Malmgren H, Wallin A (2015). Valid and efficient manual estimates of intracranial volume from magnetic resonance images. Bmc Med Imaging.

[34] Cerri S, Greve DN, Hoopes A, Lundell H, Siebner HR, Muhlau M, Van Leemput K (2023). An open-source tool for longitudinal whole-brain and white matter lesion segmentation. Neuroimage Clin.

[35] Puonti O, Iglesias JE, Van Leemput K (2016). Fast and sequence-adaptive whole-brain segmentation using parametric Bayesian modeling. Neuroimage.

[36] Gronenschild EH, Habets P, Jacobs HI, Mengelers R, Rozendaal N, van Os J, Marcelis M (2012). The effects of FreeSurfer version, workstation type, and Macintosh operating system version on anatomical volume and cortical thickness measurements. PLoS ONE.

[37] Smith SM, Jenkinson M, Woolrich MW, Beckmann CF, Behrens TEJ, Johansen-Berg H, Bannister PR, De Luca M, Drobnjak I, Flitney DE, Niazy RK, Saunders J, Vickers J, Zhang YY, De Stefano N, Brady JM, Matthews PM (2004). Advances in functional and structural MR image analysis and implementation as FSL. Neuroimage.

[38] Andersson JLR, Skare S, Ashburner J (2003). How to correct susceptibility distortions in spin-echo echo-planar images: application to diffusion tensor imaging. Neuroimage.

[39] Andersson JLR, Sotiropoulos SN (2016). An integrated approach to correction for off-resonance effects and subject movement in diffusion MR imaging. Neuroimage.

[40] Jbabdi S, Sotiropoulos SN, Savio AM, Graña M, Behrens TE (2012). Model-based analysis of multishell diffusion MR data for tractography: how to get over fitting problems. Magn Reson Med.

[41] Hernández M, Guerrero GD, Cecilia JM, García JM, Inuggi A, Jbabdi S, Behrens TE, Sotiropoulos SN (2013). Accelerating fibre orientation estimation from diffusion weighted magnetic resonance imaging using GPUs. PLoS ONE.

[42] Behrens TE, Berg HJ, Jbabdi S, Rushworth MF, Woolrich MW (2007). Probabilistic diffusion tractography with multiple fibre orientations: What can we gain?. Neuroimage.

[43] Behrens TE, Woolrich MW, Jenkinson M, Johansen-Berg H, Nunes RG, Clare S, Matthews PM, Brady JM, Smith SM (2003). Characterization and propagation of uncertainty in diffusion-weighted MR imaging. Magn Reson Med.

[44] Zhang H, Yushkevich PA, Rueckert D, Gee JC (2007) Unbiased white matter atlas construction using diffusion tensor images. In: International conference on medical image computing and computer-assisted intervention, 2007. Springer, pp 211-218 10.1007/978-3-540-75759-7_26 18044571

[45] Zhang H, Yushkevich PA, Alexander DC, Gee JC (2006). Deformable registration of diffusion tensor MR images with explicit orientation optimization. Med Image Anal.

[46] Wang Y, Shen Y, Liu D, Li G, Guo Z, Fan Y, Niu Y (2017). Evaluations of diffusion tensor image registration based on fiber tractography. Biomed Eng Online.

[47] Keihaninejad S, Zhang H, Ryan NS, Malone IB, Modat M, Cardoso MJ, Cash DM, Fox NC, Ourselin S (2013). An unbiased longitudinal analysis framework for tracking white matter changes using diffusion tensor imaging with application to Alzheimer's disease. Neuroimage.

[48] Chan K, Marques JP (2019) Susceptibility mapping pipeline tool for phase images. In: Paper Presented at the Proc. 27th Annual Meeting of the ISMRM, Montreal, Canada

[49] Li W, Avram AV, Wu B, Xiao X, Liu C (2014). Integrated Laplacian-based phase unwrapping and background phase removal for quantitative susceptibility mapping. NMR Biomed.

[50] Wood TCW (2018). QUIT: QUantitative Imaging Tools. J Open Source Softw.

[51] Smith A (1973). Symbol digit modalities test.

[52] Nauta IM, Bertens D, van Dam M, Huiskamp M, Driessen S, Geurts J, Uitdehaag B, Fasotti L, Hulst HE, de Jong BA, Klein M (2022). Performance validity in outpatients with multiple sclerosis and cognitive complaints. Mult Scler.

[53] Lechner-Scott J, Kappos L, Hofman M, Polman CH, Ronner H, Montalban X, Tintore M, Frontoni M, Buttinelli C, Amato MP, Bartolozzi ML, Versavel M, Dahlke F, Kapp JF, Gibberd R (2003). Can the expanded disability status scale be assessed by telephone?. Mult Scler.

[54] Cohen JA, Cutter GR, Fischer JS, Goodman AD, Heidenreich FR, Jak AJ, Kniker JE, Kooijmans MF, Lull JM, Sandrock AW, Simon JH, Simonian NA, Whitaker JN (2001). Use of the multiple sclerosis functional composite as an outcome measure in a phase 3 clinical trial. Arch Neurol.

[55] Cadavid D, Cohen JA, Freedman MS, Goldman MD, Hartung HP, Havrdova E, Jeffery D, Kapoor R, Miller A, Sellebjerg F, Kinch D, Lee S, Shang S, Mikol D (2017). The EDSS-Plus, an improved endpoint for disability progression in secondary progressive multiple sclerosis. Mult Scler.

[56] Dayan M, Hurtado Rua SM, Monohan E, Fujimoto K, Pandya S, LoCastro EM, Vartanian T, Nguyen TD, Raj A, Gauthier SA (2017). MRI analysis of white matter myelin water content in multiple sclerosis: a novel approach applied to finding correlates of cortical thinning. Front Neurosci.

[57] Steenwijk MD, Geurts JJ, Daams M, Tijms BM, Wink AM, Balk LJ, Tewarie PK, Uitdehaag BM, Barkhof F, Vrenken H, Pouwels PJ (2016). Cortical atrophy patterns in multiple sclerosis are non-random and clinically relevant. Brain.

[58] Cagol A, Schaedelin S, Barakovic M, Benkert P, Todea RA, Rahmanzadeh R, Galbusera R, Lu PJ, Weigel M, Melie-Garcia L, Ruberte E, Siebenborn N, Battaglini M, Radue EW, Yaldizli O, Oechtering J, Sinnecker T, Lorscheider J, Fischer-Barnicol B, Muller S, Achtnichts L, Vehoff J, Disanto G, Findling O, Chan A, Salmen A, Pot C, Bridel C, Zecca C, Derfuss T, Lieb JM, Remonda L, Wagner F, Vargas MI, Du Pasquier R, Lalive PH, Pravata E, Weber J, Cattin PC, Gobbi C, Leppert D, Kappos L, Kuhle J, Granziera C (2022). Association of brain atrophy with disease progression independent of relapse activity in patients with relapsing multiple sclerosis. JAMA Neurol.

[59] Kiljan S, Preziosa P, Jonkman LE, van de Berg WD, Twisk J, Pouwels PJ, Schenk GJ, Rocca MA, Filippi M, Geurts JJ, Steenwijk MD (2021). Cortical axonal loss is associated with both gray matter demyelination and white matter tract pathology in progressive multiple sclerosis: Evidence from a combined MRI-histopathology study. Mult Scler.

[60] Bussas M, Grahl S, Pongratz V, Berthele A, Gasperi C, Andlauer T, Gaser C, Kirschke JS, Wiestler B, Zimmer C, Hemmer B, Muhlau M (2022). Gray matter atrophy in relapsing-remitting multiple sclerosis is associated with white matter lesions in connecting fibers. Mult Scler.

[61] Weeda MM, Pruis IJ, Westerveld ASR, Brouwer I, Bellenberg B, Barkhof F, Vrenken H, Lukas C, Schneider R, Pouwels PJW (2020). Damage in the thalamocortical tracts is associated with subsequent thalamus atrophy in early multiple sclerosis. Front Neurol.

[62] Carolus K, Fuchs TA, Bergsland N, Ramasamy D, Tran H, Uher T, Horakova D, Vaneckova M, Havrdova E, Benedict RHB, Zivadinov R, Dwyer MG (2022). Time course of lesion-induced atrophy in multiple sclerosis. J Neurol.

[63] Schoonheim MM, Pinter D, Prouskas SE, Broeders TA, Pirpamer L, Khalil M, Ropele S, Uitdehaag BM, Barkhof F, Enzinger C, Geurts JJ (2022). Disability in multiple sclerosis is related to thalamic connectivity and cortical network atrophy. Mult Scler.

[64] Amiri H, de Sitter A, Bendfeldt K, Battaglini M, Gandini Wheeler-Kingshott CAM, Calabrese M, Geurts JJG, Rocca MA, Sastre-Garriga J, Enzinger C, de Stefano N, Filippi M, Rovira A, Barkhof F, Vrenken H, Group MS (2018). Urgent challenges in quantification and interpretation of brain grey matter atrophy in individual MS patients using MRI. Neuroimage Clin.

